# Modulation of auxin and cytokinin responses by early steps of the phenylpropanoid pathway

**DOI:** 10.1186/s12870-018-1477-0

**Published:** 2018-11-12

**Authors:** Jasmina Kurepa, Timothy E. Shull, Sumudu S. Karunadasa, Jan A. Smalle

**Affiliations:** Department of Plant and Soil Sciences, College of Agriculture, Food and Environment, University of Kentucky, Lexington, KY 40546-0236 USA

**Keywords:** Auxin, Cytokinin, F-box proteins, Growth promotion, Phenylpropanoid biosynthesis

## Abstract

**Background:**

The phenylpropanoid pathway is responsible for the synthesis of numerous compounds important for plant growth and responses to the environment. In the first committed step of phenylpropanoid biosynthesis, the enzyme phenylalanine ammonia-lyase (PAL) deaminates L-phenylalanine into *trans*-cinnamic acid that is then converted into *p*-coumaric acid by cinnamate-4-hydroxylase (C4H). Recent studies showed that the Kelch repeat F-box (KFB) protein family of ubiquitin ligases control phenylpropanoid biosynthesis by promoting the proteolysis of PAL. However, this ubiquitin ligase family, alternatively named Kiss Me Deadly (KMD), was also implicated in cytokinin signaling as it was shown to promote the degradation of type-B ARRs, including the key response activator ARR1. Considering that ubiquitin ligases typically have narrow target specificity, this dual targeting of structurally and functionally unrelated proteins appeared unusual.

**Results:**

Here we show that the KFBs indeed target PAL but not ARR1. Moreover, we show that changes in early phenylpropanoid biosynthesis alter cytokinin sensitivity – as reported earlier - but that the previously documented cytokinin growth response changes are primarily the result of altered auxin signaling. We found that reduced PAL accumulation decreased, whereas the loss of C4H function increased the strength of the auxin response. The combined loss of function of both enzymes led to a decrease in auxin sensitivity, indicating that metabolic events upstream of C4H control auxin sensitivity. This auxin/phenylpropanoid interaction impacts both shoot and root development and revealed an auxin-dependent stimulatory effect of *trans*-cinnamic acid feeding on leaf expansion and thus biomass accumulation.

**Conclusions:**

Collectively, our results show that auxin-regulated plant growth is fine-tuned by early steps in phenylpropanoid biosynthesis and suggest that metabolites accumulating upstream of the C4H step impact the auxin response mechanism.

**Electronic supplementary material:**

The online version of this article (10.1186/s12870-018-1477-0) contains supplementary material, which is available to authorized users.

## Background

Auxins are plant hormones that control key aspects of plant development, including the development of shoot and root meristems and cell expansion [1–3]. The auxin response pathway includes a repression relief mechanism wherein auxin promotes the degradation of AUX/IAA proteins that repress auxin responses by inhibiting the activity of the auxin response factors (ARFs), which act as transcriptional regulators of the auxin response [4, 5]. Auxin acts as a molecular glue and promotes the interaction between the AUX/IAAs and the SCF^TIR1/AFBs^ E3 ligases which commences the degradation of AUX/IAAs by the 26S proteasome [6, 7].

The link between the phenylpropanoid (PP) pathway and auxin responses has already been investigated. For example, naringenin, an early intermediate of the flavonoid branch of the PP pathway, was classified as an auxin transport inhibitor whereas the PP 3,4-(methylenedioxy)cinnamic acid was shown to interfere with auxin efflux [8, 9]. PP biosynthesis starts with L-phenylalanine that is converted into *trans*-cinnamic acid (*t*-CA) by phenylalanine ammonia lyase (PAL). *t*-CA can be converted to *cis*-cinnamic acid (*c*-CA) by light and this photoisomer has been shown to inhibit auxin transport [10, 11]. In the next step of the PP pathway, *t*-CA is converted to *p*-coumaric acid by the cytochrome P450-dependent monooxygenase cinnamate-4-hydroxylase (C4H). The reaction catalyzed by C4H marks the end of the early steps of the PP pathway and represents the pathway branching point as *p*-coumaric acid can be diverted towards the synthesis of a number of metabolite classes including lignins and flavonoids. Arabidopsis mutants that are defective in specific steps of flavonoid biosynthesis also show auxin-related developmental phenotypes [12].

PAL is the first committed enzyme of the PP pathway and its activity is regulated by environmental and endogenous signals at multiple levels [13]. At the post-translational level, the abundance of PAL isozymes is attuned to metabolic needs by the ubiquitin/proteasome pathway [14]. In Arabidopsis, PAL degradation is governed by the SCF type E3 ligases in which the target-specific component, the F-box protein called Kelch Repeat F-Box (KFB), is encoded by four genes [14]. The *KFB* genes are differentially expressed and control PAL levels in response to developmental and environmental changes. This family of ubiquitin ligases, alternatively named Kiss Me Deadly (KMD), was also shown to promote the degradation of key transcriptional activators of the cytokinin response, the type-B ARR family members ARR1 and ARR12. The *KMD/KFB* genes are down-regulated by the cytokinin signal and thus are thought to be a feed-forward mechanism that enhances the cytokinin response [15].

Cytokinins are plant growth regulators that control many agriculturally important processes, including the initiation and development of meristems and the timing of leaf senescence [16]. The cytokinin response pathway consists of a two-component signaling mechanism that involves a sequence of phosphotransfer reactions. In Arabidopsis, cytokinins are perceived by a family of three histidine kinase receptors that autophosphorylate upon binding with the hormone. The phosphoryl group is then transferred to histidine phosphotransfer proteins that in turn phosphorylate members of two functionally opposite classes of response regulators (ARRs), the response-promoting type-B ARRs and the response-inhibiting type-A ARRs. When phosphorylated, the type-B ARRs became activated and transcriptionally regulate the expression of primary cytokinin response genes. Both type-A and type-B ARRs are encoded by large gene families. Among the type-B ARRs, the *ARR1*, *ARR10* and *ARR12* genes are preeminent because their combined loss of function leads to a strong cytokinin insensitivity and severe growth reduction [17, 18].

The finding that KMD/KFBs target two sets of structurally and functionally unrelated proteins was surprising because it implies that KMD/KFBs contain two different target interaction domains and that they simultaneously control a hormone signaling pathway in addition to a secondary metabolite pathway. Here we show that the KMD/KFBs do not control the stability of the type-B ARR member ARR1 but are indeed involved in the proteasome-dependent degradation of PAL enzymes. However, we confirm the previous finding that the KMD/KFBs modulate the root growth response to cytokinin and demonstrate that this effect on cytokinin responses is a result of changes in auxin signaling. We show that loss of function of both PAL and C4H alters the response to auxin, but in an opposite manner which indicates that the observed modulation of auxin signaling is the result of metabolic changes downstream of PAL and upstream of the C4H step in the PP pathway. We also show that the product of PAL, *t*-CA, or its derivative(s) enhances auxin signaling and promotes auxin-dependent leaf expansion.

## Results

### PAL and the cytokinin response

To independently test the role of KMD/KFBs in cytokinin signaling, we generated 35S promoter-driven overexpression (OE) lines using the full-length *KMD1*/*KFB20* (At1g80440) cDNA. Earlier studies revealed that *KMD1/KFB20 *OE lines are dwarfed and that the extent of growth retardation is positively correlated with the expression level of the transgene [14, 15]**.** Indeed, 34 lines out of 52 lines we generated were also dwarfed. Both severe cytokinin resistance and disruption of the general PP pathway leads to dwarfism [18, 19]. Thus, this phenotype of the OE plants is not a diagnostic for alteration of the function of either cytokinin signaling or PP biosynthesis. Because the PP biosynthesis and cytokinin response pathways are not directly linked, we attempted to distinguish between the growth inhibition resulting from reduced PP levels and growth inhibition induced by reduced cytokinin signaling by feeding severely dwarfed *KMD1/KFB20* OE lines with PP pathway intermediates.

We grew wild-type and OE plants on media containing different concentrations of either *t*-CA, *p*-coumaric acid, *p*-coumaraldehyde, caffeic acid or quercetin. Dose-response curves are shown in Additional file 1: Figure S1. For the wild type, the feeding experiments with different doses of *t*-CA show that *t*-CA is growth promoting at low concentrations and growth inhibitory and anthocyanin inducing at high concentrations (Additional file 1: Figure S1a-c). Growth on media supplemented with *p*-coumaric acid and *p*-coumaraldehyde did not significantly change the size of the wild-type plants (Additional file 1: Figure S1d,e). Caffeic acid and quercetin treatments also did not significantly impact wild-type growth at lower doses, but they caused growth inhibition at higher doses (Additional file 1: Figure S1f, g).

**Fig. 1:**
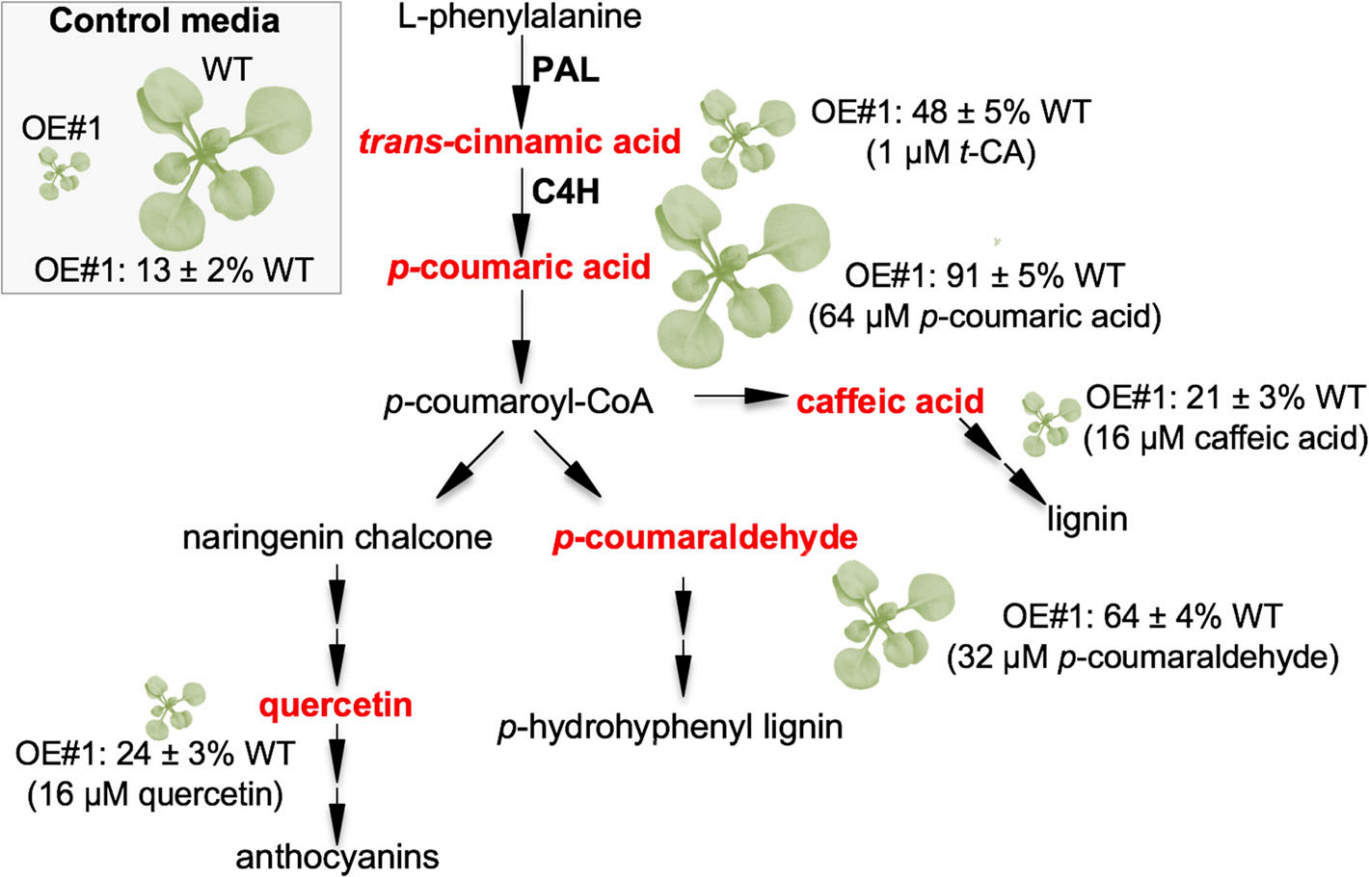
Impact of phenylpropanoid (PP) intermediates on the growth of *35S::KMD1/KFB20* (OE#1) plants. Simplified scheme of the PP biosynthetic pathway showing (in red) the PP intermediates used for feeding experiments, relative differences in rosette sizes of the OE#1 plants and fresh weight (FW) changes in OE#1 plants after 18 days of growth on MS/2 media supplemented with the specified PP intermediates. The illustration of relative size and the FW difference between the wild-type (WT) and OE#1 plants grown on control medium is presented in the shaded insert on the left-hand side. The mean fresh weights of treated OE#1 plants ± SD (*n* ≥ 12) are presented relative to the weight of the wild-type (WT) plants grown on control medium. The concentrations of the PP intermediates in the MS/2 medium for which the data are shown is noted in parenthesis. PAL, phenylalanine ammonia-lyase; C4H, cinnamic acid 4-hydroxylase

The effects of feeding with different PP intermediates differed between the wild-type and the OE#1 plants (Fig. 1, Additional file 1: Figure S1c-g). In Fig. 1, we have summarized to what extent the different PP intermediates complemented the dwarfism of the KMD1/KFB20 OE#1 plants. When grown on control medium, OE#1 plants were only 13 ± 2% the size of wild-type plants (Fig. 1). However, when grown on 1 μM *t*-CA media the size of OE#1 plants increased to 48 ± 5% of the untreated wild-type (Fig. 1). This growth-promoting effect of low doses of *t*-CA was stronger in the OE#1 line than in the wild type. For example, whereas the fresh weight of wild-type plants grown on media with 1 μM *t*-CA increased 1.41 ± 0.2 fold compared to plants grown on control media, the fresh weight of the OE#1 plants grown under the same conditions increased 3.5 ± 0.3 fold (Additional file 1: Figure S1c). Growth on media supplemented with *p*-coumaric acid and *p*-coumaraldehyde, which did not affect the size of the wild-type plants, led to a size increase in OE#1 which exceeded that measured for OE#1 plants grown on *t*-CA (Fig. 1). For example, the size of the OE#1 plants reached 91 ± 5% and 64 ± 4% of the untreated wild-type after 64 μM *p*-coumaric acid and 32 μM *p*-coumaraldehyde treatments, respectively (Fig. 1, Additional file 1: Figure S1d, e). In OE#1 plants, both quercetin and caffeic acid promoted growth at lower doses but to a lesser extent than *t*-CA, *p*-coumaric acid and *p*-coumaraldehyde (Fig. 1 and Additional file 1: Figure S1f, g). Both quercetin and caffeic acid were growth inhibitory at higher doses (Fig. 1 and Additional file 1: Figure S1f, g).

These results prompted us to reach two conclusions. First, because *t*-CA was the only compound that led to a size increase in both the wild-type and OE plants, we concluded that this metabolite has a general growth-promoting effect. Second, since feeding with metabolites of the PP pathway lead to a partial rescue, it is more likely that the primary reason for the dwarfed phenotype of the OE plants is reduced PP biosynthesis than reduced cytokinin action.

**Fig. 2:**
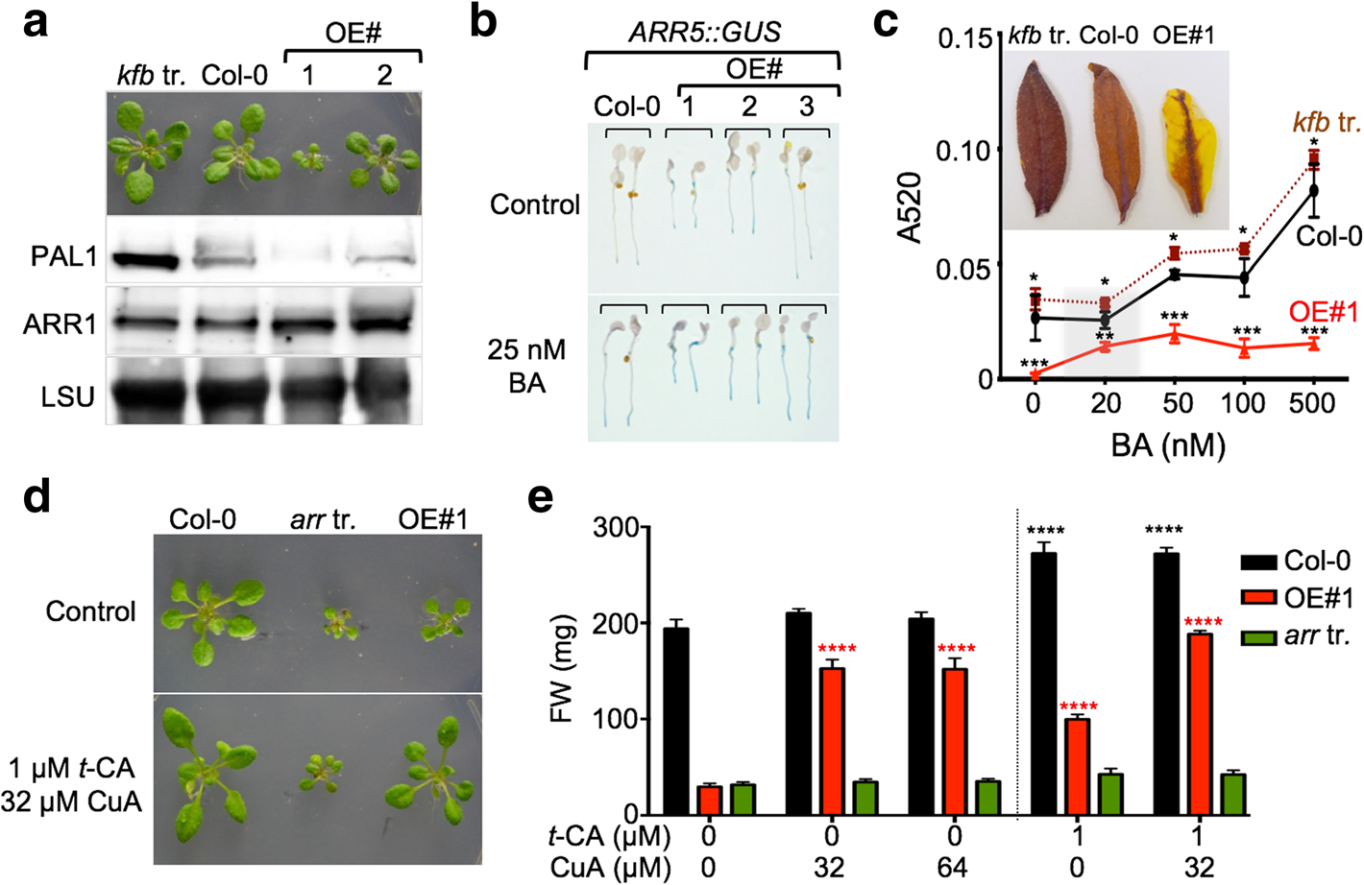
KMD1/KFB20 targets PAL and not ARR1 for proteasomal degradation. **a** Rosettes of 14-day-old plants are shown above the representative immunoblots to underline the correlations of rosette size and protein accumulation level. Rosettes of two independent *35S::KMD1/KFB20* (OE) lines are shown. The *kfb* tr. refers to the *kfb20–1 kfb1–1 kfb50–1* triple mutant. LSU, large subunit of RuBisCO is a loading control. **b** GUS activity in 4-day-old seedlings treated with 25 nM benzyladenine (BA) for 4 h prior to GUS staining. Two seedlings per line are shown. **c** Anthocyanin accumulation in 12-day-old plants is presented as the absolute absorbance of the methanolic extract at 520 nm (A520) per ten plants. Data are shown as mean ± SD (*n* ≥ 3). The significance of the difference of anthocyanin levels between Col-0 and the *kfb* triple mutant and between Col-0 and OE#1 for each treatment is noted (*, *P* < 0.05; **, *P* < 0.01; ***. *P* < 0.001; two-way ANOVA with Bonferroni’s multiple comparisons test). Insert illustrates the accumulation of anthocyanins in representative rosette leaves of 60-day-old plants. **d** Effect of *t*-CA and p-coumaric acid (CuA) on the growth of OE#1 and the *arr1–3 arr10–1 arr12–1* triple (*arr* tr.) mutant. Plants were grown on MS/2 media containing the denoted doses of *t*-CA and CuA for 18 days. **e** Statistical analyses of the effect of *t*-CA and p-coumaric acid (CuA) on the fresh weight (FW) of plants shown in **d**. Data are presented as mean ± SD (*n* ≥ 12 pools of 8 plants).). The significance of the difference between the control and the treated samples is noted for each line (****, *P* < 0.0001; two-way ANOVA with Bonferroni’s multiple comparisons test)

To further explore our second conclusion, we compared PAL and ARR1 abundance in the triple *kfb* mutant (*kfb1–1 kfb201–1 kfb501–1*) and two OE lines that differed in the strength of the dwarf phenotype (Fig. 2a). If KMD/KFBs are involved in the proteasome-dependent degradation of PAL, we expect to see an accumulation of PAL proteins in the triple *kfb* mutant and a phenotype-strength dependent reduction of PAL levels in the OE dwarfed lines. Immunoblotting analyses with anti-PAL1 antibodies confirmed this pattern of PAL accumulation and showed that while the PAL1 levels were 3 ± 0.4- fold higher in the mutant compared to wild type, PAL1 levels were reduced to ~ 10% and ~ 40% of the wild type in the OE lines (Fig. 2a). These results are in agreement with the previous study [14]. On the other hand, the ARR1 levels did not change as expected if KMD/KFBs are involved in ARR1 degradation: ARR1 did not accumulate in the triple mutant (1.1 ± 0.2 of the wild type) and its levels were not lower in the OE lines compared to the wild type. In fact, ARR1 levels were 1.8 ± 0.2- and 1.9 ± 0.3-fold higher in the OE#1 and OE#2 lines, respectively (Fig. 2a). We concluded that KMD/KFBs are indeed involved in the proteasome-dependent degradation of PAL and not in targeted proteolysis of ARR1.

An increase in the abundance of the cytokinin response activator ARR1 is expected to elicit increased cytokinin responses [20, 21]. To test if that holds true for *KMD1/KFB20* OE plants, we introduced the *35S::KMD1/KFB20* transgene into the cytokinin-inducible *ARR5::GUS* reporter line and treated a set of independent dwarfed double homozygous seedlings with the synthetic cytokinin benzyladenine (BA). The expression of *ARR5::GUS* in these double transgenic lines was indeed enhanced compared to the wild type both in untreated and BA-treated seedlings, as expected from a line with an increased ARR1 activity (Fig. 2b).

It was suggested in an earlier study that analogously to triple type-B *ARR* and triple cytokinin receptor knockout lines, the *KMD1/KFB20* OE lines are dwarfed due to their strong cytokinin resistance [15, 17]. Another phenotype of the severe cytokinin resistant lines is that they accumulate anthocyanins, which seems paradoxical because cytokinins are known inducers of anthocyanin biosynthesis [17, 22]. However, anthocyanin biosynthesis is regulated by a number of internal and external cues and an increased anthocyanin biosynthesis is often a result of the combined action of different inducing signals [23]. It has been suggested that the main cause of the anthocyanin hyperaccumulation in strong cytokinin resistant mutants is their increased sensitivity to light [17, 23]. The effect of cytokinin treatments on anthocyanin accumulation, therefore, can be viewed as another distinguishing characteristic between cytokinin resistance and alterations in PP pathway, so we measured the anthocyanin content in the KMD1-related lines treated with BA (Fig. 2c). In contrast to cytokinin resistant lines, OE#1 plants have low anthocyanin levels, which is expected if PP biosynthesis is compromised (Fig. 2c). Despite having low PAL levels (Fig. 2a), the KMD1/KFB20 OE#1 plants still responded to cytokinin by increasing anthocyanin biosynthesis (Fig. 2c). Moreover, the cytokinin-dependent induction of anthocyanin biosynthesis in OE#1 occurred at a lower dose of BA compared to the wild type, which provided another example of cytokinin hypersensitivity of plants overexpressing KMD1/KFB20. As expected, the anthocyanin levels in OE#1 did not reach wild-type levels independent of the BA dose used in the assay. The anthocyanin levels in the *kfb* triple mutant were higher than those of the wild type, but the dose-response curve had the same wild-type shape (Fig. 2c). These differences in anthocyanin accumulation patterns were clearly visible in senescing leaves (Fig. 2c).

Final confirmation that KMD/KFBs are involved in proteolysis of PAL but not ARR1 was obtained by comparing the growth responses of a strong cytokinin resistant mutant and the strong KMD1/KFB20 OE line OE#1 to PP pathway intermediates. It was previously suggested that the severe growth inhibition seen in the strong KMD1/KFB20 OE lines is mechanistically similar to the growth inhibition of the strong cytokinin resistant triple mutant *arr1–3 arr10–5 arr12–1*: both sets of lines were thought to be dwarfed as a result of reduced type-B ARR activity. If this is correct, then the growth of both *arr1–3 arr10–5 arr12–1* and KMD1/KFB20 OE plants should be similarly affected by PP pathway intermediates. However, whereas OE#1 plants reached 97 ± 2% of the untreated wild-type size on media containing both *t*-CA and *p*-coumaric acid, the *arr1–3 arr10–5 arr12–1* plants remained dwarfed and their increase in size was comparable to that of the increase observed for the wild type grown on *t*-CA and *p*-coumaric acid (40 ± 3% and 34 ± 15%, for wild type and triple *arr* mutant, respectively; Fig. 2d, e). Therefore, it is highly unlikely that the same mechanism that affects growth is operational in both the *KMD1/KFB20* OE plants and the triple *arr* mutant.

### C4H and the cytokinin response

**Fig. 3:**
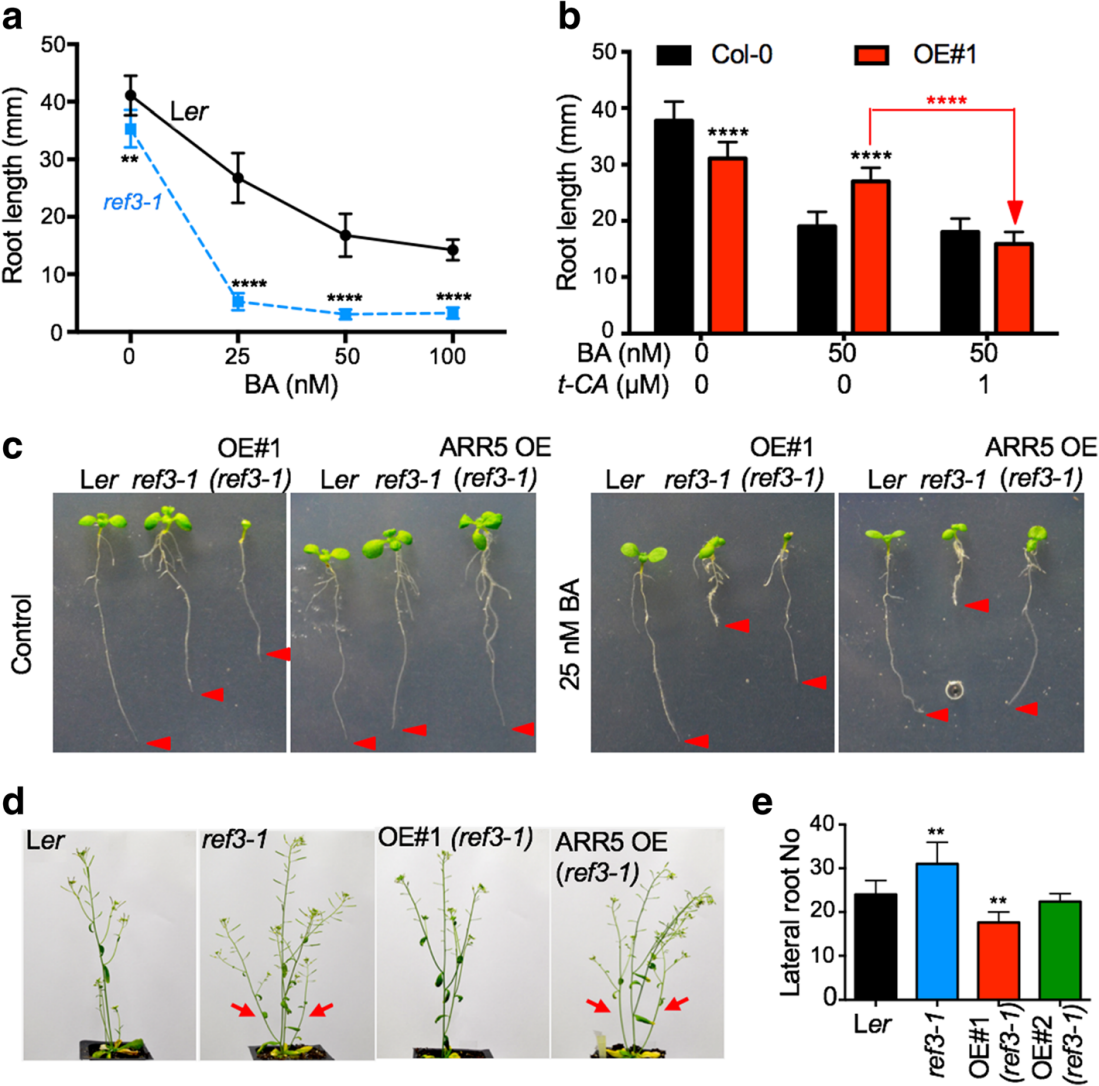
The *ref3* mutants are cytokinin hypersensitive. **a** Increased sensitivity of the strong *ref3–1* mutant (in L*er* background) in the cytokinin-induced root growth inhibition assay. Five-day-old seedlings were transferred to control or BA plates and root lengths were measured after 6 days of growth. Data are presented as absolute root length ± SD (n ≥ 12). The significance of the difference between the lengths of L*er* and *ref3–1* roots for each treatment is noted (** *P* < 0.01, ****, *P* < 0.0001; two-way ANOVA with Bonferroni’s multiple comparisons test). **b** *t*-CA-dependent restoration of wild-type cytokinin sensitivity of *35S::KMD1/KFB20* (OE#1) in root growth assays. Five-day-old seedlings were transferred from MS/2 plates to plates with the denoted doses of BA or BA and *t*-CA. The length of the primary roots was measured after 6 days of growth. Data are presented as absolute root length ± SD (n ≥ 12). The significance of the difference between the root lengths measured for Col-0 and OE#1 plants for each treatment is noted in black (****, *P* < 0.0001) and the significance of the difference between BA and combined BA and *t-*CA treatments of OE#1 plants is highlighted in red (two-way ANOVA with Bonferroni’s multiple comparisons test). ****, *P* < 0.0001 (one-way ANOVA with Bonferroni’s multiple comparisons test). **c** Introduction of *35S::ARR5* and of *35S::KMD1/KFB20* transgenes in *ref3–1* suppresses the enhanced cytokinin growth response of *ref3–1*. Plants were grown on vertical plates with or without BA and photographed after 6 days of growth. Arrowheads mark the root tips. **d** Overexpression of KMD1/KFB20 and not of ARR5 restores the increased shoot branching phenotype of *ref3–1.* Plants were grown on soil for 38 days in continuous light. Arrows are pointing to the lateral shoots. **e** Overexpression of KMD1/KFB20 restores the lateral root phenotype in *ref3–1.* Plants were grown on vertical plates for 14 days. Two independent *35S::KMD1/KFB20* transformed *ref3–1* lines are shown (OE#1 and OE#2). Data are presented as mean ± SD (*n* ≥ 10). The significance of the difference between the wild type and other lines is presented (**, *P* < 0.01; one-way ANOVA with Tukey’s post-test).

In two hallmark cytokinin response assays, type-A *ARR* expression (Fig. 2b) and anthocyanin level analyses (Fig. 2c), KMD1/KFB20 OE plants showed cytokinin hypersensitivity. A third archetypical cytokinin response assay is the root elongation assay. In this assay, as has been previously reported, KMD1/KFB20 OE plants showed decreased sensitivity to cytokinin, (Additional file 1: Figure S2a and [15]). To explain this finding, we hypothesized that a PP intermediate is required for the wild-type cytokinin root elongation response and tested this hypothesis using a genetic approach. We analyzed the cytokinin sensitivity of the *ref3* mutants, loss-of-function mutants of C4H that catalyzes the step immediately downstream of PAL [24]. Cytokinin dose-response treatments showed that *ref3* mutants are cytokinin hypersensitive (Fig. 3a and Additional file 1: Figure S2). This suggested that a PP intermediate or derivative that accumulates in *ref3* and is depleted in KMD1/KFB20 OE plants is essential for the wild-type root growth response to cytokinin. The obvious candidate was *t*-CA, so we tested whether reduced cytokinin sensitivity of OE#1 plants in root assays is restored to wild-type levels in combined BA/*t*-CA treatments. Indeed, feeding OE#1 seedlings with *t*-CA reverted the cytokinin sensitivity to the wild-type level (Fig. 3b).

Two of the more prominent visible phenotypes of *ref3* plants are increased shoot branching and increased lateral root formation ([24] and Fig. 3 d, e). Cytokinins promote lateral bud outgrowth and inhibit lateral root formation [25, 26]. Therefore, whereas increased shoot branching in *ref3* plants could be caused by cytokinin hypersensitivity, the increased lateral root formation is the opposite of what one would expect in a hypersensitive mutant. To understand this apparent contradiction, we separately introduced two transgenes into the *ref3–1* mutant line: *35S::ARR5*, which overexpresses the cytokinin response inhibitor ARR5 and thus causes cytokinin resistance, and *35S::KMD1/KFB20*, which allows us to test if the developmental changes in *ref3* plants are caused by a decreased accumulation of intermediates downstream of C4H or the accumulation of intermediates upstream of C4H. Cytokinin root growth response assays revealed that both *35S::ARR5* and *35S::KMD1/KFB20* transgenes suppressed the enhanced cytokinin growth response of *ref3* seedlings (Fig. 3c). However, only *35S::KMD1/KFB20* suppressed the increased shoot branching and the increased lateral root phenotype of the *ref3* mutant (Fig. 3d, e). Therefore, these two *ref3* phenotypes are not a result of altered cytokinin signaling but stem from the altered accumulation of metabolites synthesized upstream of the C4H step.

**Fig. 4:**
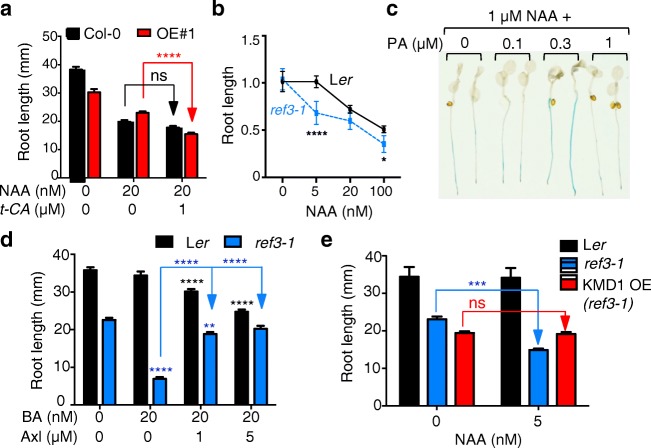
Changes in early PP biosynthesis alter the auxin response. **a** Decreased auxin sensitivity of OE#1 plants is reversed by *t*-CA. Five-day-old plants were transferred to vertical plates with the auxin 1-naphthaleneacetic acid (NAA), *t*-CA or both and the primary root length was measured after 6 days of growth. Data are shown as absolute root length ± SD (*n* ≥ 12). The significance of the difference between the NAA and the combined NAA and *t-*CA treatments of OE#1 plants is shown (ns, not significant and ****, *P* < 0.0001; two-way ANOVA with Bonferroni’s multiple comparisons test). **b** The *ref3–1* mutant is hypersensitive to auxin. The assay was performed as in **a**. Data are presented as relative root length with the mean of the control treatment assigned the value of 1. Error bars are SD (*n* ≥ 10) and the significance of the difference between L*er* and *ref3–1* for each treatment is shown (*, *P* < 0.05; ****, *P* < 0.0001; two-way ANOVA with Bonferroni’s multiple comparisons test). **c** Piperonylic acid (PA) treatments induce *DR5::GUS* expression in a dose-dependent manner. Four-day-old seedlings were co-treated with NAA and a range of PA doses. The GUS reactions were stopped upon the visible accumulation of the GUS product in the 0.3 μM PA-treated seedlings (i.e., before blue color formation in the NAA-treated control seedlings) to allow visualization of the differences in *DR5::GUS* expression. **d** Cytokinin hypersensitivity of *ref3–1* is blocked by the auxin response inhibitor auxinole (Axl). The assay was performed as in **a**. Significant differences between treated and control samples for each line is noted (in black for the wild type and in blue for the mutant) as well as the significance of the difference between the BA and the combined BA and Axl treatments of OE#1 plants (**, *P* < 0.01; ****, *P* < 0.0001; two-way ANOVA with Bonferroni’s multiple comparisons test). **e** Overexpression of KMD1/KFB20 is sufficient to suppress auxin hypersensitivity of the *ref3–1* mutant*.* The assay was performed as in **a**. The significance of the difference between the control and NAA-treated plants is shown (ns, not significant and ***, *P* < 0.001; two-way ANOVA with Bonferroni’s multiple comparisons test)

### The phenylpropanoid pathway and the auxin response

Cytokinin regulates root growth together with auxin [27]. Thus, it was possible that the altered cytokinin root growth responses of *35S::KMD1/KFB20* transgenic lines and *ref3* mutants are caused by a change in auxin sensitivity. Indeed, the auxin root growth response assay showed that OE#1 seedlings were less sensitive to auxin and their sensitivity can be restored to wild-type levels by feeding with *t*-CA (Fig. 4a). On the other hand, *ref3–1* was more sensitive to auxin (Fig. 4b). This suggested that similar to cytokinin responses, the altered auxin responses in KMD1/KFB20 OE plants and in *ref3* mutants are caused by changes in the accumulation of metabolites synthesized upstream of C4H.

Next, we used the C4H inhibitor piperonylic acid (PA) to test the expression of the cytokinin primary response reporter *ARR5::GUS* and of the auxin primary response reporter *DR5::GUS*. Whereas PA treatments did not alter the cytokinin-dependent induction of *ARR5::GUS* (Additional file 1: Figure S3), the expression of the auxin-induced *DR5::GUS* was affected in a dose-responsive manner, with the maximal induction of *DR5::GUS* recorded at 0.3 μM PA (Fig. 4c). These results supported our conclusion that cytokinin hypersensitivity of *ref3* mutants is a response to a change in auxin sensitivity. To confirm that, we added the auxin response inhibitor auxinole to the cytokinin root elongation growth response assay and compared the responses of the wild type and *ref3–1* (Fig. 4d). Whereas the root length of wild-type plants was not affected by the low concentration of 20 nM BA, root elongation was inhibited when auxinole was included in the medium (Fig. 4d). In contrast, *ref3–1* responded with strong root growth inhibition to the same BA dose, but this hypersensitive growth response was suppressed by auxinole, indicating that this enhanced cytokinin response is indeed caused by enhanced auxin signaling (Fig. 4d).

Finally, to test if changes in metabolite accumulation upstream of C4H caused the altered auxin response, we transformed *ref3–1* with the *35S::KMD1/KFB20* transgene and tested the auxin sensitivity of the resulting line. Overexpression of KMD1/KFB20 was sufficient to suppress the auxin hypersensitivity of the *ref3* mutant (Fig. 4e).

### *t*-CA, auxin and cell expansion

One of the major effects of auxin is the promotion of cell expansion at lower doses and inhibition of cell expansion at higher doses [28, 29]. We showed that when growing plants on media supplemented with PP intermediates, only *t*-CA led to an increase in the size of the wild-type plants (Fig. 1). *t*-CA feeding led to an increase in both fresh weight and dry weight and this positive effect on growth was observed for both the Col-0 and L*er* ecotypes (Additional file 1: Figure S1c and S4).

**Fig. 5:**
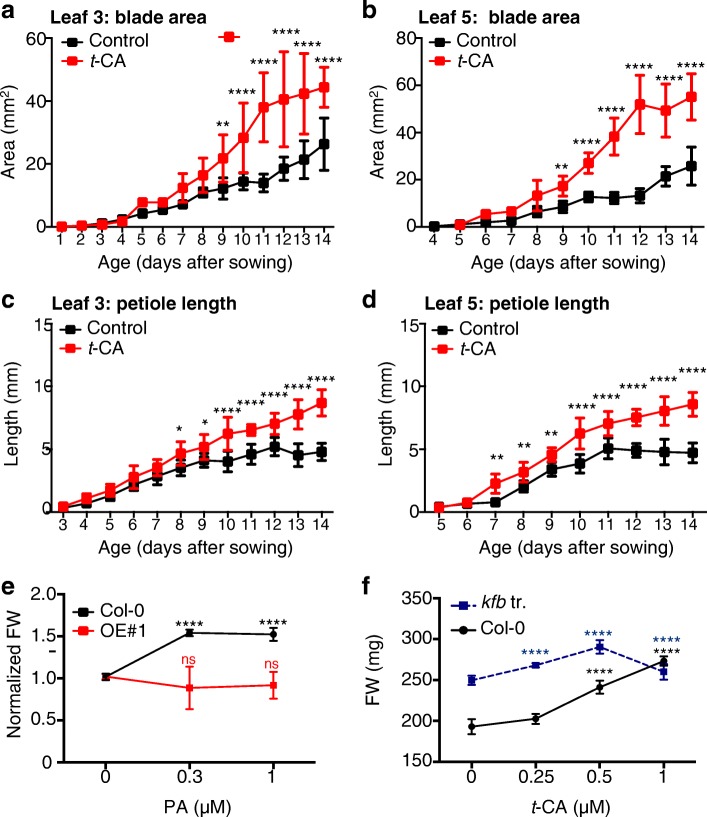
*t*-CA promotes leaf expansion and petiole elongation. **a**-**d** Kinematic growth analysis of the third (L3) and the fifth (L5) leaf of Col-0 plants grown on control media or media with 1 μM *t*-CA. Error bars represent mean ± SD (*n* ≥ 8). The significant differences between *t-*CA-treated and control samples for each day is noted (**, *P* < 0.01; ****, *P* < 0.0001; two-way ANOVA with Bonferroni’s multiple comparisons test). **e** Piperonylic acid (PA) treatments differentially affect growth of the wild-type and KMD1/KFB20 overexpression (OE#1) plants. Plants were grown on MS/2 media supplemented with the denoted doses of PA for 18 days. Due to the size difference of the rosettes of the tested lines, the fresh weight (FW) was presented as normalized mean ± SD (*n* ≥ 6, each sample containing 6 seedlings) with the mean FW for each line grown on control media being assigned the value of 1. The significance of the difference between the control and treatments is noted for each line (ns, not significant, ****, *P* < 0.0001; two-way ANOVA with Bonferroni’s multiple comparisons test). **f** Size increase in *kfb20–1 kfb1–1 kfb50–*1 (*kfb* tr.) plants treated with the denoted doses of *t*-CA. Experimental setup and data analyses are as described in **e**. The significance of the difference between treated and untreated plants for each line is shown (****, *P* < 0.0001; two-way ANOVA with Bonferroni’s multiple comparisons test)

**Fig. 6:**
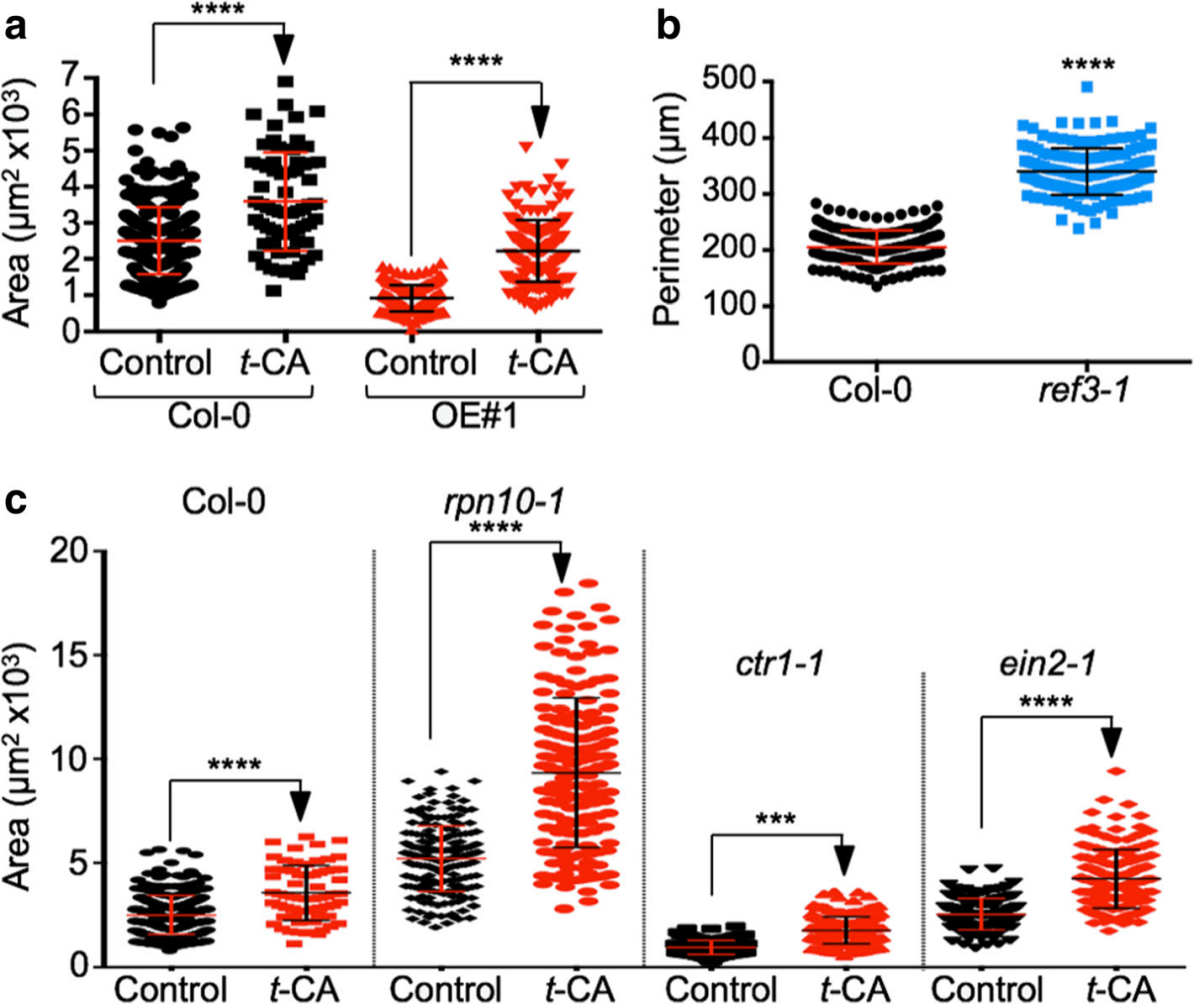
*t*-CA induces cell expansion. **a** Area of cotyledonary epidermal cells in the 14-day-old wild-type and KMD1/KFB20 OE#1 plants grown on control media or media with 1 μM *t*-CA. Data are mean ± SD (*n* ≥ 150 cells from at least five cotyledons). ****, *P* < 0.0001 (two-way ANOVA with Bonferroni’s multiple comparisons test). **b** Perimeter of the cotyledonary mesophyll cells of plants grown on control media for 14 days. ****, *P* < 0.0001 (two-tailed *t*-test, *n* > 150). **c** Area of cotyledonary epidermal cells in 14-day-old wild-type plants and proteasome (*rpn10–1)* and ethylene (*ctr1–1* and *ein2–1*) mutants. Data are mean ± SD (*n* ≥ 100 cells from at least six cotyledons per line). ***, *P* < 0.001 and ****, *P* < 0.0001 (two-way ANOVA with Bonferroni’s multiple comparisons test)

To understand the link between *t*-CA-dependent growth promotion and auxin action, we analyzed the growth kinetics and cell sizes of plants grown on different media (Figs. 5 and 6). Kinematic analyses of wild-type plants grown on *t*-CA-supplemented media showed that *t*-CA promotes leaf expansion and petiole elongation (Fig. 5a-d). *t-*CA treatment also increased the duration of the petiole elongation phase compared to the untreated control (Fig. 5c, d). To exclude the possibility that the increased growth of *t-*CA fed plants was due to an accumulation of intermediates synthesized in later steps of the PP pathway, we analyzed the growth response to the C4H inhibitor PA. In the wild type, treatment with low doses of PA promoted rosette growth to a similar level as *t*-CA (49 ± 8% at 1 μM PA). In contrast to the wild type, the size of the OE#1 plants was not influenced by PA, suggesting that the PA effect depends on the PAL-dependent synthesis of *t*-CA (Fig. 5e). The OE#1 seedlings were also less responsive to PA treatment in a root growth response assay: two wild-type PA responses, inhibition of root elongation and increased lateral root formation, were suppressed in the OE#1 seedlings (Additional file 1: Figure S5a, b). Analyses of the size of triple *kfb* mutant plants showed that when grown on control media, 18-day-old mutant plants were 41 ± 8% larger than the wild type (Fig. 5f). The size increase of the triple *kfb* mutant can be phenocopied by growing wild-type plants on 1 μM *t*-CA (Fig. 5f). Compared to the wild type, a lower dose of *t*-CA already promoted growth of the triple *kfb* mutant (e.g., 0.25 μM) and the 1 μM dose was growth inhibitory, suggesting that the triple *kfb* mutant is hypersensitive to *t*-CA. This suggested that the size increase of the triple *kfb* mutant was indeed caused by increased *t*-CA synthesis. Taken together, these results suggested that the growth-promotive effect of *t*-CA is caused by either *t*-CA itself or a compound(s) derived from *t*-CA upstream of the C4H step.

To investigate the basis of the *t-*CA-stimulated growth increase, we measured epidermal cell sizes using the tissue printing technique (Fig. 6a). Cells of 1 μM *t*-CA treated wild-type plants were 45 ± 10% larger than the cells of the untreated plants, which is an enlargement that corresponds well with the size increase of rosettes. The cells of the untreated OE#1 plants were smaller than of the untreated wild type (37 ± 1 1% wild-type size) and their size increased by *t*-CA feeding surpassing the increase seen in the wild type (150 ± 8%; Fig. 6a). To determine if the cell size reduction in OE#1 plants is caused by changes in PP intermediate accumulation upstream or downstream of C4H, we analyzed the cell sizes of *ref3* mutants. If the cell size reduction in OE#1 plants is caused by the decreased accumulation of metabolites downstream of C4H, then it was expected that *ref3* plants should also have smaller cells. If, however, the OE#1 cell size decrease is caused by the decreased accumulation of metabolites upstream of C4H, then *ref3* cells are expected to be larger because the loss of C4H function is expected to cause increased accumulation of metabolites upstream of C4H. We were unable to determine epidermal cell sizes in *ref3–1* cotyledons using the tissue printing technique because of their extreme epinasty, which has been described previously [24]. However, analyses of cotyledon mesophyll cells did reveal a 65 ± 18% increase compared to the wild type (Fig. 6b).

To integrate the growth-promotive effect of *t*-CA (or its derivative(s)) with other processes that are known to lead to an increase in cell expansion, we tested the effect of *t*-CA feeding on the strong 26S proteasome mutant *rpn10–1* and two ethylene mutants, *ein2–1* and *ctr1–1* (Fig. 6c). Loss of function of 26S proteasome regulatory particle (RP) subunits, such as RPN10, was previously shown to cause increased leaf cell expansion accompanied by decreased cell division [30]. Growth on *t*-CA-supplemented media caused a further increase in cell expansion in *rpn10–1* (Fig. 6c). *t*-CA also induced cell expansion both in the *ctr1–1* mutant, which has a strong constitutive ethylene response and a dwarf phenotype caused by a reduction in cell size [31], and in the strong ethylene insensitive mutant *ein2–1*, which was shown to have larger palisade cells [32] but not larger epidermal cells (Fig. 6c). These results suggested that the mechanism of *t*-CA-dependent regulation of cell expansion does not involve ethylene and that it acts in parallel to effects induced by reduced 26S proteasome-dependent proteolysis.

**Fig. 7:**
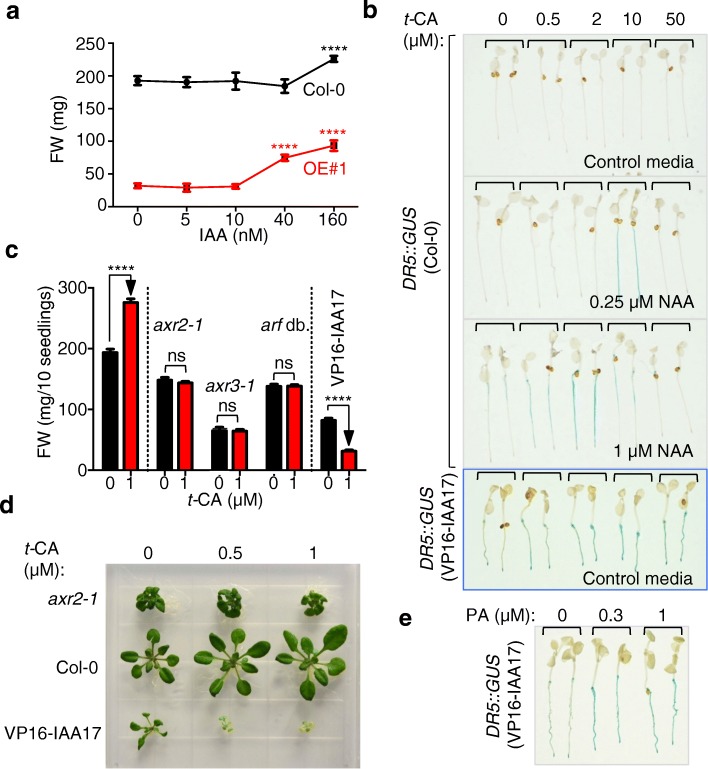
*t*-CA promotes the auxin response. **a** OE#1 dwarfism is partially rescued by indole-3-acetic acid **(**IAA) in a rosette growth response assay. Plants were grown on media supplemented with the denoted doses of IAA for 18 days. Pools of ten plants were measured and data are presented as mean ± SD (*n* ≥ 10). The significance of the difference between the treated and untreated plants for each line is shown (****, *P* < 0.0001; two-way ANOVA with Bonferroni’s multiple comparisons test). **b** *t*-CA enhances auxin signaling. Five-day-old plants expressing *DR5::GUS* in either Col-0 or *35S::VP16-IAA17mImII (*VP16-IAA17*)* background were treated for 5 h with the denoted doses of NAA and *t*-CA. The GUS reactions were stopped upon visible accumulation of blue color in the NAA and *t*-CA co-treated seedlings (i.e., before blue color accumulation in the respective NAA-treated controls). Similarly, the GUS reactions were stopped upon visible accumulation of blue color in the *t-*CA treated *35S::VP16-IAA17mImII DR5::GUS* seedlings (i.e., before blue accumulation in the untreated control). **c** Auxin resistant mutants (*axr2–1*, *axr3–1* and *arf7–1 arf19–1* (*arf* db.)) are insensitive whereas a line with increased auxin response (VP16-IAA17*)* is hypersensitive to 1 μM *t*-CA in a rosette growth response assay. The fresh weight (FW) of plants was measured in pools of ten plants after 18 days of growth. Data are presented as mean ± SD (*n* ≥ 8). ns, not significant; ****, *P* < 0.0001 (one-way ANOVA with Bonferroni’s multiple comparisons test). **d** Rosettes of plants grown on media supplemented with the denoted doses of *t-*CA for 21 days. **e** Piperonylic acid (PA) enhances expression of the *DR5::GUS* reporter in the *35S::VP16-IAA17mImII (*VP16-IAA17*)* background. Five-day-old plants were treated for 5 h with the denoted doses of PA. The GUS reactions were stopped upon visible accumulation of blue color in the PA-treated *35S::VP16-IAA17mImII (*VP16-IAA17*) DR5::GUS* seedlings (i.e., before blue accumulation in the untreated control)

Because of the link between *t*-CA or its derivatives and auxin signaling (Fig. 4), we next investigated whether auxin-dependent regulation of cell expansion underlies *t*-CA-promoted leaf growth. Prolonged growth on a medium supplemented with a low dose-range of indole-3-acetic acid (IAA; 5 to 160 nM) showed that the highest tested dose induced a 17 ± 5% increase in the rosette size of the wild-type and a 2.6 ± 0.2 fold increase in OE#1 (Fig. 7a). This suggested that the dwarfism of the OE#1 line was in part caused by a decrease in auxin action and that this was complemented by increasing the auxin concentration. We concluded that *t*-CA-dependent growth promotion is linked to auxin regulation of plant cell expansion implying that *t*-CA and/or its derivatives also promote this auxin response.

To reaffirm that *t*-CA or its derivatives promote auxin responses, we analyzed the effect of *t*-CA on the expression of the auxin-inducible *DR5::GUS* reporter and on the development of lines with altered auxin sensitivity (Fig. 7b, d). For the first experiment, we have used the synthetic auxin 1-naphthaleneacetic acid (NAA) instead of the natural auxin IAA. Because NAA is known to more readily diffuse into plant cells compared to IAA, NAA has been the auxin of choice for short-term response assays such as the analyses of *DR5::GUS* reporter expression [33, 34]. Whereas *t*-CA alone did not induce the expression of *DR5::GUS*, it enhanced the effect of NAA on *DR5::GUS* expression in a complex dose-dependent manner (Fig. 7b). The minimal auxin dose that induces *DR5::GUS* expression was 250 nM NAA (Additional file 1: Figure S6a). The addition of *t*-CA to 250 nM NAA enhanced the expression of *DR5::GUS*, but only at higher doses of *t*-CA, such as 10 μM (Fig. 7b). When the *t*-CA dose-response experiments were repeated using 1 μM NAA, the effective dose of *t*-CA was lower compared to the dose that enhanced the expression of *DR5::GUS* at 250 nM NAA (Fig. 7b). These results prove that *t*-CA (and/or *t-*CA derivative(s)) indeed promote auxin signaling and suggest that *t*-CA leads to plant cell expansion by modulating auxin responses.

Finally, we analyzed the *t*-CA-induced growth response of auxin resistant mutants *axr2* and *axr3* in which the auxin signaling repressor proteins IAA7 and IAA17 are stabilized, and an *arf* mutant which carries loss-of-function mutations in auxin response-regulating *ARF* genes. Strikingly, all tested mutants (*axr2–1*, *axr3–1* and the *arf7–1 arf19–1* double mutant) were *t*-CA insensitive in the growth promotion assay (Fig. 7c,d). Treatments with high concentrations of auxin are growth inhibitory and this can be mimicked by expression of the *35S::VP16-IAA17mImII* transgene that causes a strong increase in auxin signaling [35]. The plants of this transgenic line responded to the *t*-CA treatment with a further reduction in growth, confirming that *t*-CA enhances auxin action also when auxin responses become growth inhibitory (Fig. 7c, d). Next, we introduced the *DR5::GUS* reporter into *35S::VP16-IAA17mImII* background and tested the effect of *t*-CA on its expression. As expected, the *DR5::GUS* reporter was constitutively expressed in the *35S::VP16-IAA17mImII* background (Additional file 1: Figure 6b). The expression of the *DR5::GUS* reporter was further enhanced both by *t*-CA and PA treatments (Fig. 7b, e). We concluded that *t*-CA or its derivative(s) are de facto modulators of auxin signaling.

## Discussion

Although the overall conclusion of this study is that early steps in the phenylpropanoid pathway are important modulators of auxin-regulated plant growth, this work has also prompted a number of other discussion points. The first discussion point relates to our analyses of the function of the KMD/KFB ubiquitin ligase family. A major step in ubiquitin-dependent proteolysis is the interaction of a target protein with a ubiquitin E3 ligase that promotes the attachment of a polyubiquitin chain to one or more lysine residues within the target [36]. A key feature of a ubiquitin ligase is that it binds its target protein in a highly specific manner and it typically contains a distinct target-interaction domain. It is not surprising, therefore, that the Arabidopsis genome encodes for numerous ubiquitin ligases, each having binding affinity for one target or a highly related family of target proteins [37]. This complexity and multiplicity of different E3 ligases reflect the fact that the abundance of numerous proteins is controlled by the ubiquitin-proteasome system, often in response to specific environmental or endogenous signals [38]. It was therefore unusual that the KMD/KFBs were reported to target two structurally and functionally unrelated classes of proteins, the PAL enzymes and the type-B ARRs transcription factors [14, 15]. In addition, the results of interactomics projects, such as PSICQUIC-View [39], confirmed the binding of KMD/KFBs to PAL, but reported no interactions between KMD/KFBs and type-B ARRs. Here, we show that the endogenous ARR1 protein, one of the essential type-B ARRs previously shown to be under KMD control, is not targeted for proteasome-dependent degradation by KMDs and that PAL is indeed a legitimate target for this F-box protein family. Because we have previously shown that the stability control of tagged ARR1 versions differs from the stability control of the endogenous ARR1 [20], we believe that the use of tagged versions of type-B ARRs is the underlying reason for this misidentification of KMD targets.

A second discussion point involves the interactions of the PP pathway with auxin and cytokinin. Whereas we could not confirm any role for the KMD/KFBs in the control of ARR1 stability, our data support the reported findings that *KFB20/KMD1* overexpression causes a decrease in cytokinin sensitivity in a root elongation response assay [15]. However, we show that the decreased sensitivity is not caused by a defect in cytokinin signaling and that in fact, *KMD1/KFB20* overexpression causes an increase in ARR1 abundance and consequently, leads to cytokinin hypersensitivity. It follows that the decreased cytokinin root growth response is caused by a change acting in parallel or downstream of the cytokinin signaling pathway. It has been shown that the effect of cytokinins on root growth involves auxin regulation and that auxin resistance impairs cytokinin-regulated root development [40, 41]. In this study, we showed that *KMD1/KFB20* overexpression indeed causes decreased sensitivity to auxin whereas the loss of function of C4H in *ref3* mutants led to auxin and cytokinin hypersensitivity in growth response assays and enhanced auxin signaling. Moreover, we showed that co-treatment with the auxin response inhibitor auxinole suppressed the cytokinin hypersensitivity of *ref3* seedlings, which confirmed our hypothesis that the changes in cytokinin growth responses caused by defects in early PP biosynthesis are caused by changes in auxin signaling. The simplest explanation for these findings is that the decreased auxin sensitivity of KMD/KFB overexpression lines is caused by decreased *t*-CA synthesis and that the increased sensitivity of *ref3* is caused by an increased accumulation of *t*-CA or its derivative(s). Indeed, decreased PAL levels caused by *KMD1/KFB20* overexpression suppressed the *ref3* hypersensitivities to auxin and cytokinin, which aligns with our hypothesis that these *ref3* hypersensitive phenotypes are caused by the increased accumulation of a metabolite upstream of the C4H step.

The third question raised is the identity of the early PP metabolite that regulates auxin responses. It is currently unknown whether *ref3* mutants have increased *t-*CA content [24]. However, it was shown earlier that *t*-CA does not necessarily accumulate when the function of C4H is compromised [42]. PAL, which catalyzes the first committed step into the PP pathway, is under negative feedback regulation by *t*-CA both on the transcriptional and enzymatic activity levels and blocking the pathway at C4H leads to both a product feedback-dependent reduction of *t*-CA synthesis rate and a redirection of carbon flow into branches that are used less under normal conditions [42]. Two examples of metabolic redirection of carbon flow after C4H inhibition are known: the accumulation of cinnamoylmalate, which is typically not detectable in untreated wild-type plants [24] and an increased synthesis of salicylic acid [43]. Therefore, it is possible that instead of *t-*CA itself, one or more *t*-CA derivatives are the actual enhancers of the auxin response.

The next question to be addressed is how and at what level does auxin signaling and responses interact with PP biosynthesis. A previous study showed that C4H inhibition leads to an increase in auxin biosynthesis that, together with a change in auxin transport, brought about the developmental phenotype of C4H loss-of-function mutants [9]. Whereas increased auxin biosynthesis does not explain the auxin hypersensitivity of *ref3* mutants, changes in auxin transport can lead to alterations of auxin sensitivity [44]. A candidate metabolite that can alter auxin transport is *cis*-cinnamic acid (*c-*CA), a photoisomer of *t-*CA, that was recently shown to be an auxin efflux inhibitor [10]. However, as already stated, C4H inhibition or loss of function does not necessarily lead to an increase in *t-*CA level and by extension, should not necessarily lead to an increase in *c-*CA concentration. Therefore, it remains possible that another *t-*CA derivative that accumulates upstream of C4H directly impacts the auxin signaling mechanism. The flavonoid biosynthetic pathway - one of the downstream branches of the PP pathway – was also shown to be involved in the modulation of auxin transport [12]. If the auxin hypersensitivity of *ref3* mutants was caused by the decreased accumulation of flavonoids then decreasing the PAL function in the *ref3* background would not suppress but would enhance the auxin hypersensitivity, which is not what we observed. Our results, therefore, reveal that there are multiple interaction points between auxin and the PP pathway.

The next discussion point and one of the main findings of this study is centered on the strong growth promoting effect of *t-*CA in Arabidopsis. This promotive effect was, however, detected only when low concentrations (e.g., 0.5 and 2 μM) of *t-*CA were used for treatments. The existence of a narrow dose range in which *t-*CA acts as a growth promotor after which it becomes growth inhibitory may be universal for all plants and may explain the results of previous studies that describe both positive and negative effects of *t-*CA on growth [45–47]. Here we have shown that the positive effect of *t-*CA on leaf expansion requires an intact auxin response pathway, thus further strengthening the relation between early PP biosynthesis and auxin regulation. We also concluded that the dwarfism associated with *KMD1/KFB20* overexpression is a result of the loss of two growth-promoting activities of *t-*CA: the depletion of downstream PP pathway compounds needed for growth and loss of *t*-CA-dependent promotion of auxin action.

Finally, although the primary focus of this study was the effects of early PP biosynthesis on auxin regulation, the increased ARR1 accumulation and increased cytokinin signaling in *KMD1/KFB20* overexpression plants are interesting observations that warrant further discussion. This increased sensitivity at the signaling level was however not accompanied by increased sensitivity at the growth response level. Instead, we observed a decreased sensitivity to cytokinin in a root elongation response assay and we showed that this is caused by decreased *t*-CA synthesis as this insensitivity was reversed in combined *t*-CA/BA treatments. It thus would appear that the reduction in PAL accumulation in *KMD1/KFB20* overexpression plants simultaneously causes an increase in ARR1 abundance and a change that prevents this ARR1 increase to promote the cytokinin growth response. Currently, we see two ways by which decreased PAL activity can lead to an increase in ARR1 abundance. The first possibility is that the same early PP metabolites that regulate auxin responses directly or indirectly regulate ARR1 accumulation. The second possibility is that the severe growth inhibition of *KMD1/KFB20* OE plants causes an increase in ARR1 levels by simply altering the developmental stage of cells. In this case, the increased ARR1 accumulation would reflect the developmental regulation of *ARR1* gene expression. Future research will have to address these two hypotheses and reveal if any other mechanisms are at play.

## Conclusions

Here, we have shown that changes in early PP biosynthesis alter auxin sensitivity and that these changes, in turn, alter both root and shoot development. Because the early steps in PP biosynthesis are regulated by environmental and developmental signals, our results suggest that the early steps of the PP pathway play a key role in the environmental and developmental control of plant growth.

## Methods

### Plant material

The wild-type lines used were Columbia (Col-0) and Lansberg *erecta* (L*er*) dependent on the background of the mutations analyzed. The following previously described mutants and transgenic lines were used: the *kfb20–1 kfb1–1 kfb50–*1 triple mutant [14], the *arr1–3 arr10–5 arr12–1* triple mutant [17], *ref3–1, ref3–2* and *ref3–3* [24], *ARR5::GUS* [48], *DR5::GUS* [33], *rpn10–1* [49], *ctr1–1* [31], *ein2–1* [50], *axr2–1* [51], *axr3–1* [52], *arf7–1 arf19–1* [53] and *35S::VP16-IAA17mImII* [54]*.* Except for the *kfb20–1 kfb1–1 kfb50–*1 triple mutant, *rpn10–1* and *35S:ARR5,* all other lines were obtained from the ABRC Seed Stock Center.

The following transgenes were introduced by Agrobacterium-mediated transformation into the following backgrounds: *35S::KMD1/KFB20* into the Col-0 wild type (phosphinothricin resistant), *35S::KMD1/KFB20* into *ARR5::GUS* (phosphinothricin resistant), *35S::KMD1/KFB20* into *ref3–1* (phosphinothricin resistant) and *35S::ARR5* into *ref3–1* (phosphinothricin resistant). The *35S::ARR5* construct used to generate *ARR5* overexpression lines was previously described [55]. To generate *KMD1/KFB20* overexpression lines, the full-length cDNA clone was amplified using attB-capped primers. The amplified and verified fragment was recombined by BP reaction into pDONR221 and transferred to pEarlyGate100 [56] by LR reaction using the Gateway protocols (Invitrogen). The resulting binary vector was introduced into *Agrobacterium tumefaciens* strain C58C1 (Rif-R) by triparental mating and the plants were transformed by the floral dip method [57]. The *35S::VP16-IAA17mImII DR5::GUS* line was generated by introgression of the *35S::VP16-IAA17mImII* and *DR5::GUS* transgenic lines, and subsequent selection for plants homozygous for both the *35S::VP16-IAA17mImII* developmental phenotype and GUS activity.

### Materials

The following chemicals were used for treatments: *trans*-cinnamic acid (*t-*CA; Sigma), *p*-coumaric acid (Sigma), caffeic acid (Sigma), *p*-coumaraldehyde (Sigma), quercetin (Sigma), benzyladenine (BA; Sigma), 1-naphthaleneacetic acid (NAA; Sigma), piperonylic acid (PA; Sigma) and auxinole [58, 59]. All were prepared as stock solutions in dimethylsulfoxide (DMSO, Fisher Scientific), which was used as the mock control in treatments.

### Growth conditions

Both sterile- and soil-grown plants were grown in controlled environmental growth chambers at 22 °C under continuous light at 80 μmolm^− 2^ s^− 1^. For axenic cultures, surface-sterilized and stratified seeds were sown on half-strength Murashige and Skoog medium (pH 5.7) containing 1% sucrose and 0.8% PhytoAgar (MS/2 medium). For soil growth, plants were first grown in sterile cultures and then transferred to a 1:1 mix of Miracle Grow potting soil and vermiculite. For feeding experiments, we chose to test a *t-*CA concentration range based on an earlier report that a minimal dose of 100 μM is sufficient for increasing the synthesis of lignin in soybean [60]. After initial tests, the test concentrations range for Arabidopsis was adjusted to 0 to 125 μM *t*-CA and the doses used for other PP intermediates were then chosen in a similar range.

### Antibody production and immunoblotting analyses

The Arabidopsis ARR1 antibody has been described [20]. Monospecific anti-PAL rabbit antibodies were generated (Pacific Immunology, Ramona, CA) against two internal peptides of PAL1 (At2g37040): Cys-TSHRRTKNGVALQKE (amino acids 126–140) and KVLTTGVNGELHPSRFC (555–571). After affinity purification and specificity testing, the antibodies raised against PAL1 (126–140) were used. Protein extraction and immunoblotting analyses were performed as previously described [61]. The secondary antibodies used (horseradish peroxidase-conjugated anti-rabbit IgG goat antibodies) were obtained from SantaCruz Biotechnology. Immunoblots were developed using SuperSignal West Femto substrate (Thermo-Pierce) using a ChemiDoc™ XRS molecular imager (Bio-Rad). The signal intensities of two independent immunoblots were measured using QuantityOne software (Bio-Rad).

### GUS staining

For histochemical GUS analyses, seedlings were transferred to a staining buffer solution (10 mM Na_2_EDTA, 100 mM NaH_2_PO_4_, 0.1% Triton X-100) that contained 1 mg/ml X-Gluc substrate. To stop the reaction and prepare for photography, seedlings were first transferred to ethanol, then to a 50% glycerol solution and were finally arranged on MS/2 plates for photography. Different incubation times were used for the GUS activity assays dependent on the aim of the experiment.

### Phenotype analyses and statistical methods

For all morphometric and kinematic analyses, five-day-old seedlings germinated and grown on MS/2 plates were transferred to fresh MS/2 plates containing the test compounds. For rosette size analysis, plants were photographed daily and the measurements were done from photographs using ImageJ software. Root length analyses were done as described [55]. For lateral root number, visible lateral roots of any length and developmental stage were counted. For anthocyanin measurements, 10 plants per replicate (3 replicates per sample) were collected after 12 days of growth on test plates, weighed and used for isolation of total flavonoids as described previously [62]. For anthocyanin content measurement, a DTX 880 multimode detector (Beckman Coulter) with 520 ± 8 nm filter was used. Cell size analyses were done either using the agarose print method or by lactophenol clearing [30]. A minimum of five cotyledons per line was used to determine cell numbers and cell sizes.

The descriptive statistics, plotting and hypothesis testing were done using Prism 6 software (GraphPad Software Inc). All data are presented as means ± SD of at least three independent experiments. When means of more than two samples were compared, we used one-way nonparametric ANOVA followed by Bonferroni’s posttest to find a significant difference between pairs of means. The significance levels, indicated by asterisks in the figures, illustrate the results of the Bonferroni’s posttest.

## Additional file

Additional file 1: **Figure S1.** Impact of PP intermediates on growth. **Figure S2.** Sensitivity of *ref3* alleles in the cytokinin root elongation growth response assay. **Figure S3.** The C4H inhibitor piperonylic acid (PA) does not alter cytokinin-induced *ARR5::GUS* expression. **Figure S4.** *t*-CA-dependent growth promotion. **Figure S5.** Piperonylic acid (PA) treatments differentially affect growth of the wild-type and KMD1/KFB20 overexpression (OE#1) plants. **Figure S6.** Expression of the auxin-inducible *DR5::GUS* reporter. (DOCX 7659 kb)

## Abbreviations

ARF: Auxin response factor
ARR: Arabidopsis response regulator
Axl: Auxinole
BA: Benzyladenine
C4H: Cinnamate-4-hydroxylase
*c*-CA: *cis*-cinnamic acid
CuA: *p*-coumaric acid
IAA: Indole-3-acetic acid
KFB: Kelch Repeat T F-Box
KMD: Kiss Me Deadly
NAA: 1-naphthaleneacetic acid
OE: Overexpression
PA: Piperonylic acid
PAL: Phenylalanine ammonia-lyase
PP: Phenylpropanoid
RP: Regulatory particle
*t*-CA: *trans*-cinnamic acid
