# Supplementary material for: Modulation of auxin and cytokinin responses by early steps of the phenylpropanoid pathway
Source: BMC Plant Biol. 2018 Nov 12;18:278. doi: 10.1186/s12870-018-1477-0 (PMC6233370; doi:10.1186/s12870-018-1477-0)
Supplement: Supplementary file 1 — Figure S1. Impact of PP intermediates on growth. Figure S2. Sensitivity of ref3 alleles in the cytokinin root elongation growth response assay. Figure S3. The C4H inhibitor piperonylic acid (PA) does not alter cytokinin-induced ARR5::GUS expression. Figure S4. t-CA-dependent growth promotion. Figure S5. Piperonylic acid (PA) treatments differentially affect growth of the wild-type and KMD1/KFB20 overexpression (OE#1) plants. Figure S6. Expression of the auxin-inducible DR5::GUS reporter. (DOCX 7659 kb) [file 12870_2018_1477_MOESM1_ESM.docx]

## Additional File 1

**Fig. S1.** Impact of PP intermediates on growth.

**a** Effects of a broad-range of *trans*-cinnamic acid (*t*-CA) doses on the growth of the wild-type (Col-0) plants. Plants were photographed after 11 days of growth.

**b** Accumulation of anthocyanins in the wild-type plants grown for 11 days on *t*-CA-supplemented media. Data are presented as mean absorbance at 520 nm (A520) ± SD (n≥6, each sample being a pool of 10 seedlings). P < **, 0.01 and ****, P < 0.0001 (one-way ANOVA with Bonferroni’s multiple comparisons test).

**c - g** Dose-response curves of the wild-type and *35S::KMD1/KFB20* plants (OE#1) grown in media containing the denoted doses of PP intermediates. The fresh weight (FW) of rosettes of 18-day-old plants was measured and the average absolute FW of the wild type grown on control media was set to 1. Data are shown as mean ± SD (n≥12). The significance of the difference between the control and treatments is noted in black for Col-0 and in red for OE#1 (*, P < 0.05; **, P < 0.01; ***, P < 0.001; ****, P < 0.0001; two-way ANOVA with Bonferroni’s multiple comparisons test). Shaded areas highlight the effective doses that augment the growth of OE#1 plants.

**Fig. S2**. Sensitivity of *ref3* alleles in the cytokinin root elongation growth response assay.

**a** Cytokinin sensitivity of roots of the OE#1 plants and plants of the weak *ref3-3* mutant (in Col-0 background). Five-day-old seedlings were transferred to control or BA plates and the length of the primary root was measured from photographs taken after 6 days of growth. Data are presented as relative root length ± SD (n≥12) with the root length of the wild type on control media assigned the value of 1. The significance of the difference for a given concentration of BA (ns, not significant, *, P < 0.05 and ****, P < 0.0001; two-way ANOVA with Bonferroni’s multiple comparisons test) are given in green for OE#1 vs. Col-0 and in red for *ref3-3* vs. Col-0.

**b** Analyses of the Col-0 background mutant *ref3-2*. The *ref3-2* mutant is the strongest mutant of the tested *ref3* allelic series and has to be maintained in the heterozygote state. Primary root length of the homozygous segregants was tested on a single BA dose that was certain to reveal the hypersensitive response based on data obtained for the *ref3-1* and *ref3-3* mutants. Data are presented as absolute root length ± SD (n≥9). The significance of the difference between treated and not treated plants (ns, not significant and ****, P < 0.0001; two-way ANOVA with Bonferroni’s multiple comparisons test) is marked in black for Col-0 and in blue for *ref3-2*.

**Fig. S3.** The C4H inhibitor piperonylic acid (PA) does not alter cytokinin-induced *ARR5::GUS* expression.

Four-day-old seedlings were co-treated with the noted doses of BA and PA. The GUS reaction was stopped upon the visible accumulation of blue color in the 25 nM BA treated seedlings. Two representative seedlings per treatment are shown.

**Fig. S4.** *t*-CA-dependent growth promotion.

**
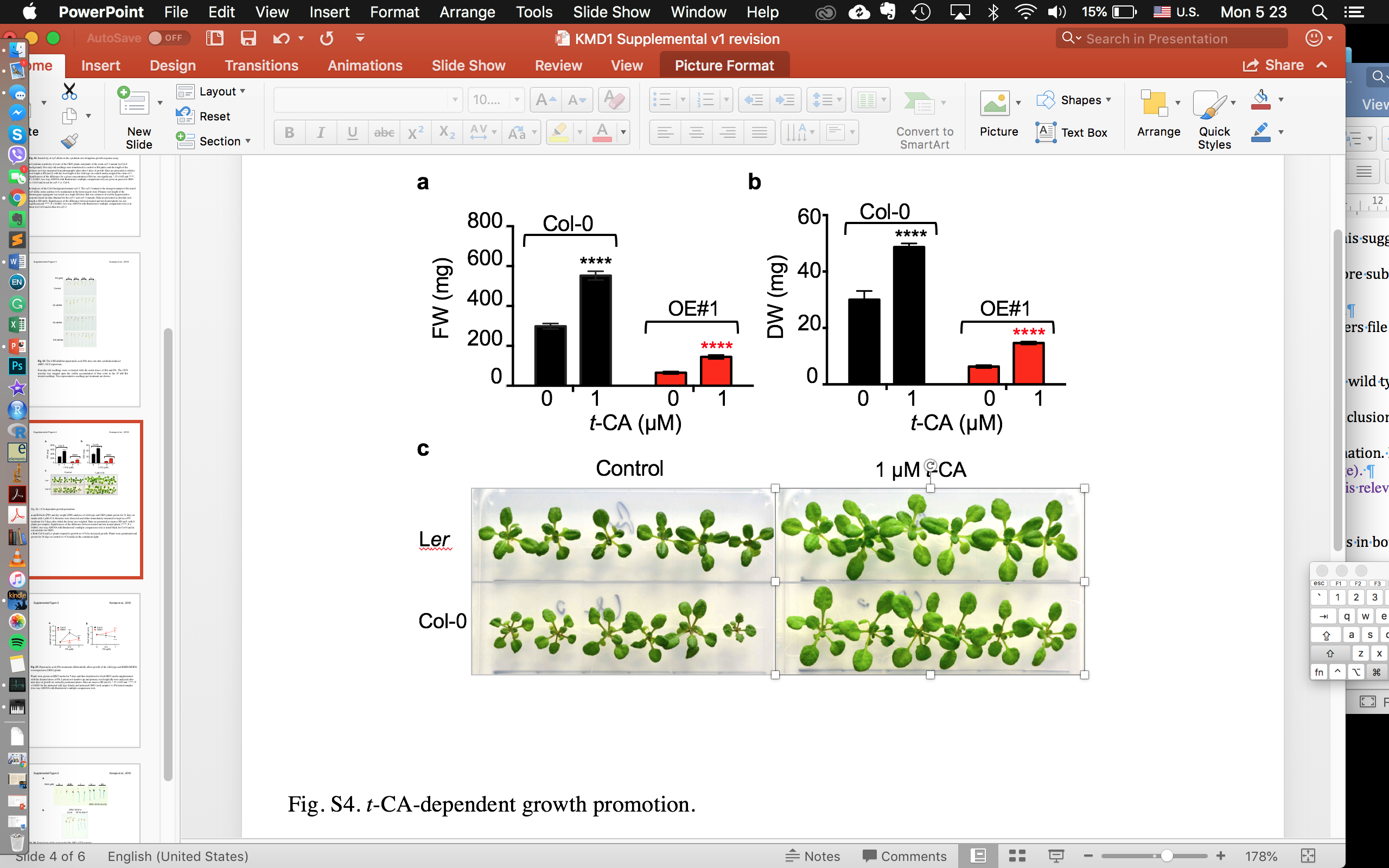
**

**a** and **b** Fresh (FW) and dry weight (DW) analyses of wild-type and OE#1 plants grown for 21 days on media with 1 µM *t*-CA. Rosettes were dissected and either immediately measured or kept in a 65ºC incubator for 5 days after which the tissue was weighed. Data are presented as mean ± SD (n≥5, with 8 plants per sample). The significance of the difference between treated and not treated plants (****, P < 0.0001; two-way ANOVA with Bonferroni’s multiple comparisons test) is marked in black for Col-0 and in red for OE#1.

**c** Both Col-0 and L*er* plants respond to the growth-promoting effect of *t*-CA. Plants were germinated and grown for 18 days on control or *t*-CA-supplemented media in continuous light.

**Fig. S5.** Piperonylic acid (PA) treatments differentially affect growth of the wild-type and KMD1/KFB20 overexpression (OE#1) plants.

Plants were grown on MS/2 media for 5 days and then transferred to fresh MS/2 media supplemented with the denoted doses of PA. Lateral root number (**a**) and primary root length (**b**) were analyzed after nine days of growth on vertically positioned plates. Data are presented as mean ± SD (n≥12). The significance of the difference between treated and not treated plants (*, P < 0.05 and ****, P < 0.0001; two-way ANOVA with Bonferroni’s multiple comparisons test) is marked in black for Col-0 and in red for OE#1.

**Fig. S6**. Expression of the auxin-inducible *DR5::GUS* reporter.


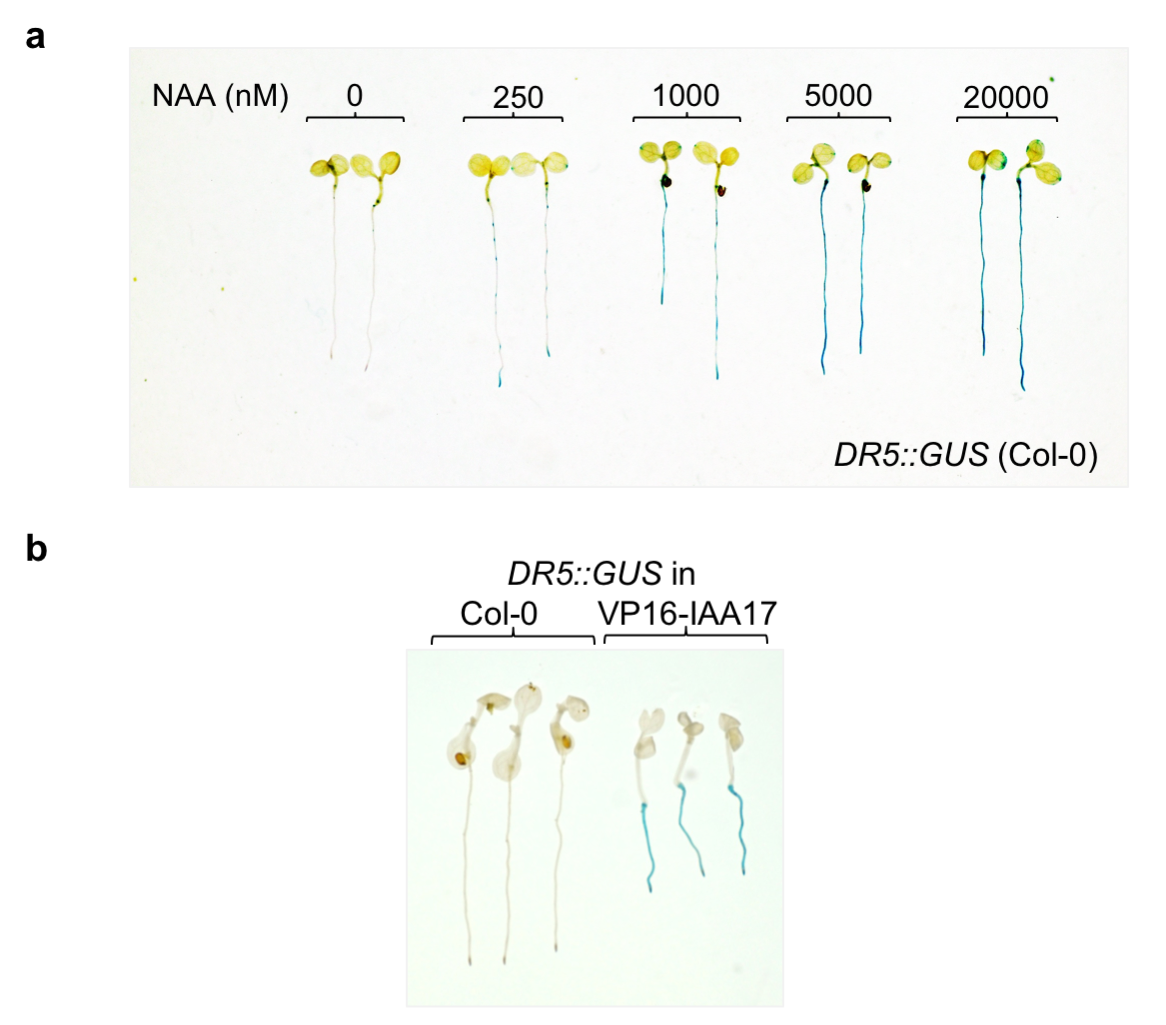


**a** *DR5::GUS* seedlings were grown on MS/2 media for 5 days and then treated with the denoted doses of NAA for 5 hrs. Two representative seedlings per dose are shown.

**b** *DR5::GUS* expression is constitutively up-regulated in the *35S::VP16-IAA17mImII* (VP16-IAA17) background. Three representative seedlings in the wild type and *35S::VP16-IAA17mImII* backgrounds are shown.
